# Cognitive Impairment in Relapsing-Remitting Multiple Sclerosis Patients with Very Mild Clinical Disability

**DOI:** 10.1155/2017/7404289

**Published:** 2017-08-15

**Authors:** S. Migliore, A. Ghazaryan, I. Simonelli, P. Pasqualetti, F. Squitieri, G. Curcio, D. Landi, M. G. Palmieri, F. Moffa, M. M. Filippi, F. Vernieri

**Affiliations:** ^1^Clinical Psychology, University Campus Bio-Medico of Rome, Rome, Italy; ^2^LIRH Foundation, Via dei Mille 41, Rome, Italy; ^3^Department of Neuroscience, Fatebenefratelli Hospital-Isola Tiberina, Rome, Italy; ^4^Service of Medical Statistics and Information Technology (SeSMIT), Fatebenefratelli Hospital-Isola Tiberina, Rome, Italy; ^5^IRCCS Casa Sollievo della Sofferenza, San Giovanni Rotondo, Italy; ^6^Department of Life, Health and Environmental Sciences, University of L'Aquila, L'Aquila, Italy; ^7^Department of Neuroscience, Policlinico “Tor Vergata”, Rome, Italy; ^8^Neurology Unit, University Campus Bio-Medico of Rome, Rome, Italy

## Abstract

Cognitive dysfunction affects 40–65% of multiple sclerosis (MS) patients and can occur in the early stages of the disease. This study aimed to explore cognitive functions by means of the Italian version of the minimal assessment of cognitive function in MS (MACFIMS) in relapsing-remitting MS (RRMS) patients with very mild clinical disability to identify the primarily involved cognitive functions. Ninety-two consecutive RRMS patients with Expanded Disability Status Scale (EDSS) scores ≤ 2.5 and forty-two healthy controls (HC) were investigated. Our results show that 51.1% of MS patients have cognitive dysfunction compared to HC. An impairment of verbal and visual memory, working memory, and executive functions was found in the RRMS group. After subgrouping RRMS by EDSS, group 1 (EDSS ≤ 1.5) showed involvement of verbal memory and executive functions; moreover, group 2 (2 ≤ EDSS ≤ 2.5) patients were also impaired in information processing speed and visual memory. Our results show that utilizing a comprehensive neuropsychological assessment, approximately half of MS patients with very mild physical disability exhibit cognitive impairment with a primary involvement of prefrontal cognitive functions. Detecting impairment of executive functions at an early clinical stage of disease could be useful to promptly enroll MS patients in targeted rehabilitation.

## 1. Introduction

Cognitive impairment (CI) is a common deficit of multiple sclerosis (MS), with prevalence rates ranging from 40 to 65% [1]. It can have a dramatic impact on a patient's quality of life, influencing role fulfilment in work as well as in social life independent of physical disability [2]. The cognitive domains mostly affected are attention, visuospatial abilities, learning and memory, information processing speed, and problem solving, while “simple” attention and essential verbal skills are not usually compromised [3, 4]. To identify cognitive impairment, scores on the single test are usually used. Recently, Migliore et al. [5] considered also the cognitive domains rather than the single tests to better identify patients with multidomain cognitive impairment. This classification may be more specific to identify MS patients with a clear cognitive impairment; in fact, patients with two tests failed in the same domain are not considered multidomain cognitively impaired.

Cognitive dysfunction can be detected even at the earliest stages of the disease [6, 7]; nevertheless, its prevalence is higher in chronic progressive patients [7]. Longitudinal studies indicate that CI, if present, progresses over time [4, 8, 9]. Moreover, CI has a prognostic value as it indicates a shifting to a progressive phase and motor impairment [8, 10]. The disease course influences cognitive performance profiles: at the very early stage of the disease, that is, in clinically isolated syndrome (CIS) patients, the main domains involved are processing speed and executive functions [7, 9], while in the relapsing-remitting MS (RRMS) course, verbal and visual memory [7, 11] is also affected. Patients with a chronic progressive course, however, tend to exhibit a more frequent, severe, and widespread CI [7, 12].

Many other clinical variables have been explored to determine which one mostly influences cognition in MS patients, and results have been controversial. In particular, CI is scarcely correlated with disease duration [8, 13]. These results may be explained by the difficulty in determining the disease onset. It should be noted, however, that patients with the same disease duration and activity may have completely different levels of physical disability. The evidence of a relationship between CI and level of physical disability is also conflicting [8]. Rao and colleagues [14] reported a slight but significant correlation between physical disability and the presence or degree of CI, whereas other studies failed to find any significant association [15]. More recently, a significant correlation of CI with physical disability was found in a heterogeneous sample of MS patients [7, 16].

Our aim was to explore cognitive function in RRMS patients with very mild clinical disability by means of an ad hoc comprehensive neuropsychological assessment (Italian version of the minimal assessment of cognitive function in multiple sclerosis (MACFIMS)) [5], to identify early affected cognitive domains regardless of disease duration. Moreover, we aimed to evaluate potential correlations among CI and clinical parameters such as Expanded Disability Status Scale (EDSS), disease duration, and neuropsychiatric features.

## 2. Materials and Methods

### 2.1. Subjects

Our study included ninety-two patients with a diagnosis of RRMS (according to McDonald's criteria) [17] and forty-two healthy controls (HC) comparable by age, sex, and education. From September 2013 to December 2014, patients were selected at the MS centers of the Neuroscience Department at San Giovanni Calibita “Fatebenefratelli” Hospital (Rome), at the Policlinico “Tor Vergata” (Rome), and at the Neurology Outpatient Clinic of Campus Bio-Medico University (Rome). We contacted the research participants (including all HC) either by mail/telephone or approaching them during their periodic clinical examinations. Considering our patients' study population, about 25% of the RRMS patients were monitored about their cognitive functioning, 25% of them were investigated for specific clinical reasons (i.e., disability evaluation, differential diagnosis of CI versus depressive disorder, suspected cognitive impairment), and about 50% were assessed as research volunteers (i.e., they had no cognitive problems). Our MS sample may be considered representative of the MS population referred to MS centers. Hospital employees (physicians, nurses, clerks, cleaners, and porters) and their relatives were included in the HC group.  Table 1 shows demographic and clinical characteristics of the MS and HC groups.

Inclusion criteria were as follows: age 18 or older, fluent in Italian, able to provide informed consent to all procedures, and EDSS ≤ 2.5 (for patients only).

Exclusion criteria were as follows: neurological disorders other than MS; psychiatric disorder other than mood, personality, or behavior change following the onset of MS; medical condition that might influence cognition; history of developmental disorder (e.g., ADHD, learning disability); history of substance or alcohol dependence or current abuse; motor or sensory deficits that might interfere with cognitive test performance; and relapse and/or corticosteroid pulse within four weeks of assessment (for patients only).

We decided not to include or exclude patients on the basis of the medication they were taking. However, none of the participants were under treatment that has a significant impact on their cognitive performance; all patients were under immunomodulant therapy (interferon or glatiramer acetate). A detailed clinical interview was performed to verify the inclusion and exclusion criteria, and each patient underwent complete neurological examinations including EDSS rating. According to Kurtzke's criteria [18], patients were separated into two different subgroups in line with EDSS: in particular, patients with EDSS ≤ 1.5 were not considered to have any disability [group 1, no physical disability (ND)], while patients with EDSS between 2 and 2.5 were considered to have very mild disability (group 2, VMD) (Table 1). We considered EDSS ≤ 2.5 a cut-off in order to investigate only the cognitive function in patients with very mild levels of disability. Each HC and MS patient signed an informed written consent (previously approved by the local ethical committee) to participate in the study.

### 2.2. Neuropsychological Assessment

Both MS patients and HC underwent an Italian version of MACFIMS [5] and neuropsychiatric questionnaires by an expert clinical neuropsychologist. Tests were administered in a standardized manner, during daytime in a quiet room and in a fixed order, in accordance with consensus panel recommendations [3, 19].

Moreover, the participants completed the Beck Depression Inventory (BDI) [20] and State-Trait Anxiety Inventory Form Y (STAI-Y) [21] to check for psychiatric comorbidity. The entire test battery required 90/100 minutes of face-to-face testing time.

Patients were diagnosed as having CI when at least two tests were found to have more than 1.5 standard deviations (SD) below the control mean, according to the proposal of Amato et al. [22]. Moreover, employing 1.5 SD, Benedict et al. [3] found a strong association between MACFIMS tests and vocational outcomes, proving that 1.5 SD is a reliable parameter to detect CI. However, we did a further analysis taking into account the 5th percentile, in order to have a more restrictive parameter for detecting CI [5].

On the basis of the number of test in the CI range, patients were classified as mildly (two tests impaired), moderately (three tests impaired), or severely affected (four or more tests impaired) [5, 12]. This classification reflects different levels of cognitive deterioration in order to highlight different severity degrees of cognitive dysfunction.

In addition to the number of tests failed, we aimed to consider the number of cognitive domains impaired, according to Migliore et al. [5]. More specifically, the domain was considered altered when at least one test in the domain had an impaired result. MS patients were considered multidomain cognitively impaired (mDCI) when at least two domains were found to be altered. In total, five cognitive domains were considered: verbal memory (CVLT total learning—CVLT TL, CVLT long-term memory—CVLT LTM), visual memory (BVMT total learning—BVMT TL, BVMT long-term memory—BVMT LTM), information processing speed (Paced Auditory Serial Addition Task—PASAT 3″ and PASAT 2″, Symbol Digit Modalities Test—SDMT), executive functions (Delis-Kaplan Executive Function System Correct Sort—DKEFS CS, DKEFS description score—DKEFS DS, and Controlled Oral Word Association Test—COWAT), and visuospatial perception (Judgment of Line Orientation—JLO). We also considered the cognitive domains rather than the single tests to better identify those patients showing a multidomain cognitive impairment. This classification may result in a greater specificity in identifying MS patients with a clear cognitive impairment. In fact, patients with two tests impaired in the same domain were not classified as multidomain cognitively impaired (mDCI).

### 2.3. Statistical Analysis

**Figure 1:**
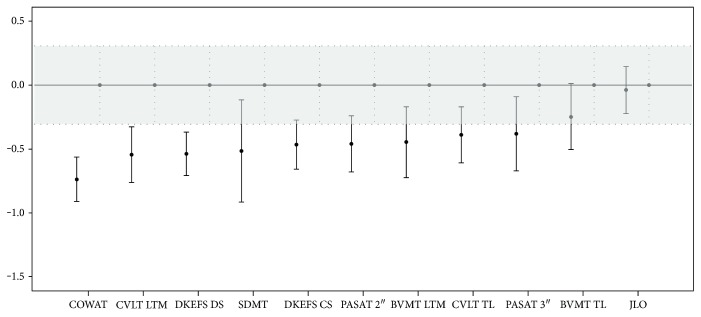
Cognitive performance pattern (*z*-score mean and 95% confidence interval) in MS patients (closed circles), compared to HC (mean set at 0, with grey band indicating 95% confidence interval). All raw scores, for each scale, were transformed into *z*-scores using the mean and the standard deviation (SD) of healthy controls. CVLT TL: California Verbal Learning Test total learning; CVLT LTM: CVLT long-term memory; BVMT TL: Brief Visuospatial Memory Test total learning; BVMT LTM: BVMT long-term memory; SDMT: Symbol Digit Modalities Test; PASAT 3″ and 2″: Paced Auditory Serial Addition Test; JLO: Judgment of Line Orientation; DKEFS CS: Delis-Kaplan Executive Function System sorting test correct sort; DEKEFS DS: DEKEFS description score; COWAT: Controlled Oral Word Association Test.

**Figure 2:**
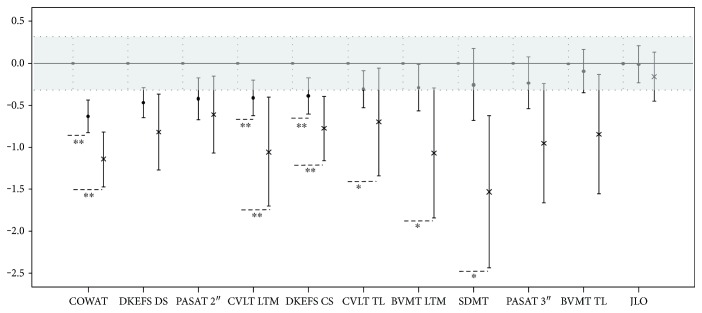
Cognitive performance pattern (*z*-score mean and 95% confidence interval) in the EDSS subgroups (closed circles for no disability; asterisk for very mild disability), compared to healthy controls (mean set at 0, with grey band indicating 95% confidence interval). All raw scores, for each scale, were transformed into *z*-scores using the mean and the standard deviation (SD) of healthy controls. CVLT TL: California Verbal Learning Test total learning; CVLT LTM: CVLT long-term memory; BVMT TL: Brief Visuospatial Memory Test total learning; BVMT LTM: BVMT long-term memory; SDMT: Symbol Digit Modalities Test; PASAT 3″ and 2″: Paced Auditory Serial Addition Test; JLO: Judgment of Line Orientation; DKEFS CS: Delis-Kaplan Executive Function System sorting test correct sort; DEKEFS DS: DEKEFS description score; COWAT: Controlled Oral Word Association Test. Post hoc comparisons: ^∗^*p* < 0.05; ^∗∗^*p* < 0.01.

Group differences regarding demographic and clinical data were assessed using parametric tests (Student's *t*-test or univariate ANOVA). Correlations among neuropsychological tests and disease duration, BDI and STAI-Y, were evaluated using Spearman's correlation coefficients. Analyses of covariance (ANCOVA) were applied to examine differences in test performance considering diagnosis and sex as factors and BDI as a covariate. To obtain neuropsychological profiles shown in Figures 1 and 2, we transformed all raw scores, for each scale, into *z*-scores using the mean and the standard deviation (SD) of healthy controls of the present study. Repeated-measures ANOVA were performed on these *z*-score variables to compare the performance of each patient group to each neuropsychological test, considering the test type as the within-subjects factor and the group as the between-subjects factor. If the assumption of sphericity was violated, Greenhouse-Geisser correction of degrees of freedom was considered.

To describe the effect size, the “Cohen's d” was calculated; it is the difference between means divided by the pooled SD, and its magnitude is assessed using the thresholds provided by Cohen [23], whereby 0.2 equates to a small effect, 0.5 equates to a medium effect, and effects larger than 0.8 correspond to large effects. Overall, a *p* value less than 0.05 was considered significant. Tukey's adjustment or, in cases of variance heterogeneity, Dunnett's adjustment was applied for post hoc comparisons. A log transformation was applied to BDI to gain a better fit to Gaussianity, to limit the dangerous effects of extreme values, and to reduce heteroscedasticity in the residuals. All statistical analyses were performed using SPSS 16.

## 3. Results

### 3.1. Comparison between MS Group and HC

Neuropsychological test scores for both MS patients and HC are shown in Table 2. According to repeated-measures ANOVA, besides the expected group effect (F(1,131) = 11.006; *p* = 0.001), the group X test type interaction (F(12,1584) = 1.963; *p* = 0.02) was also significant, indicating that the difference between the two groups changed across tests (Figure 1()). Looking separately at each test, we found that the patient group performed significantly worse than HC in verbal memory total learning (CVLT TL), long-term memory (CVLT LTM), visuospatial long-term memory (BVMT LTM), working memory in a subtest with high cognitive load (PASAT 2″), and executive functions (DKEFS CS, DKEFS DS, and COWAT). No significant differences were observed for the other neuropsychological (NP) tests.

According to the definition of CI reported above (at least two tests impaired with 1.5 SD below the control mean), 51.1% of patients were classified as impaired (out of them, 16.3% were mildly, 15.2% were moderately, and 19.6% were severely impaired). Moreover, even when considering the 5th percentile (1.645 SD below the control mean), 40.2% of patients had impaired results (out of them, 10.9% were mildly, 15.2% were moderately, and 14.1% were severely impaired). Detailed data about each NP test are reported in Table 2.

Regarding the number of impaired cognitive domains, according to the cut-off of 1.5 SD below the control mean, 43.5% of MS patients showed at least two domains compromised (out of them, 20.7% were mildly, 9.8% were moderately, and 13.1% were severely impaired). Moreover, if we consider the 5th percentile, 33.7% of patients were found compromised (out of them, 15.2% were mildly, 8.7% were moderately, and 9.8% were severely impaired).

A significant difference was also found on BDI scores comparing MS patients and HC (*p* = 0.006). ANCOVA with diagnosis and sex as factors and BDI (log scale) as a covariate was applied to compare test scores in MS patients and HC. In general, the differences between MS patients and controls still remained significant, with the exception of PASAT 2″, where the effect of depression (although marginally significant; F(1,89) = 3.69, *p* = 0.058) tempered down the difference between the two groups (see Table 4 in the Supplementary Material available online at https://doi.org/10.1155/2017/7404289). Moreover, a significant effect of sex was observed on the COWAT score (F(1,89) = 5.15, *p* = 0.026). STAI-Y did not differ between MS patients and controls. Also, disease duration did not significantly influence NP scores (all *p* values > 0.2).

### 3.2. Comparison among EDSS Subgroups and HC

Repeated-measures ANOVA revealed a significant main effect of the groups (F(2,131) = 8.632; *p* < 0.001). According to pairwise Tukey's comparisons, the controls' overall estimated mean was not significantly different from that of the ND group (*p* = 0.085) but was significantly higher than that of the VMD group (*p* < 0.001) as expected. Also ND's overall estimated mean was significantly higher than that of the VMD group (*p* = 0.015).

A significant effect of the group X test type interaction (F(df1_Greenhouse-Geisser_ = 12.25 and df2_Greenhouse-Geisser_ = 802.59) = 1.771; *p* = 0.047) was also found, indicating again that the differences among groups were dependent on the type of test (Figure 2). Separate ANOVAs were conducted for each NP subtest to evaluate intergroup differences, revealing significant differences among groups in verbal memory (CVLT TL: F(2,131) = 3.95, *p* = 0.022; CVLT LTM: F(2,131) = 8.597, *p* < 0.001), visuospatial long-term memory (BVMT LTM: F(2,131) = 3.977, *p* = 0.021), executive functions (DKEFS CS: F(2,131) = 6.441, *p* = 0.002; DKEFS DS: F(2,131) = 8.144, *p* < 0.001; and COWAT: F(2,131) = 11.195, *p* < 0.001), and information processing speed (SDMT: F(2,131) = 3.220, *p* = 0.043).

Post hoc comparisons showed that there were significant differences between HC and the ND group in verbal memory (CVLT LTM, *p* = 0.007) and executive functions (DKEFS CS, *p* = 0.014; DKEFS DS, *p* = 0.002; and COWAT, *p* = 0.002) (Table 3). HC and VMD were significantly different in verbal memory (CVLT TL, *p* = 0.045; CVLT LTM, *p* = 0.001), visuospatial long-term memory (BVMT LTM, *p* = 0.016), information processing speed (SDMT, *p* = 0.042), and executive functions (DKEFS CS, *p* = 0.005; DKEFS DS, *p* = 0.003; and COWAT, *p* < 0.001). No significant differences were observed between the ND and VMD subgroups (Table 3).

It should be noted that with such an unbalanced distribution of cases in the two subgroups (73 ND versus 19 VMDP), only large standardized effect sizes (>0.8) have enough probability (power > 80%) to be recognized as statistically significant at the defined significance threshold (0.05).

**Figure 3:**
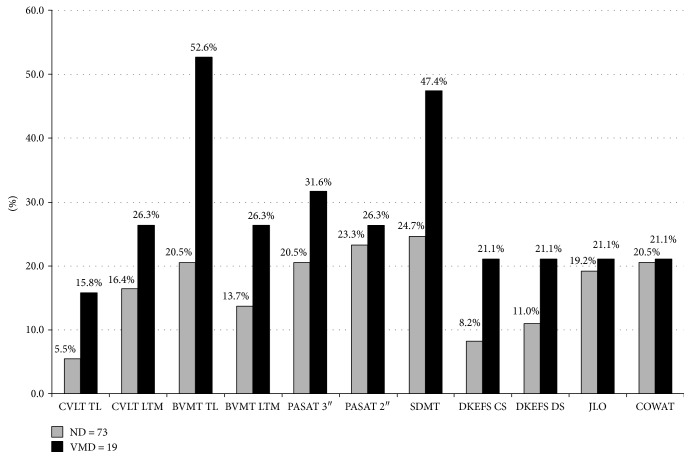
Percentage of impairment in each multiple sclerosis patient subgroup. CVLT TL: California Verbal Learning Test total learning; CVLT LTM: CVLT long-term memory; BVMT TL: Brief Visuospatial Memory Test total learning; BVMT LTM: BVMT long-term memory; SDMT: Symbol Digit Modalities Test; PASAT 3″ and 2″: Paced Auditory Serial Addition Test; JLO: Judgment of Line Orientation; DKEFS CS: Delis-Kaplan Executive Function System sorting test correct sort; DEKEFS DS: DEKEFS description score; COWAT: Controlled Oral Word Association Test. ND: no disability; VMD: very mild disability.

In Figure 3, for each subgroup considered, percentages of pathological scores (1.5 SD below the control mean) for every NP test are reported, and the VMD, with respect to ND, reflected a greater rate of cognitive impairment in almost every test considered.

## 4. Discussion

Our results demonstrate that 51.1% of our MS patients with EDSS ≤ 2.5 have cognitive dysfunction (at least two tests ≤ 1.5 SD); in particular, 16.3% had a mild (two tests failed), 15.2% had a moderate (three tests failed), and 19.6% had a severe (≥four tests failed) level of impairment. Even when we applied a more restrictive cut-off (5th percentile), 40.2% of patients were cognitively impaired. Moreover, considering the number of impaired cognitive domains, we found that 43.5% of MS patients showed at least two compromised domains. Also, by applying the 5th percentile cut-off to the number of impaired domains, we observed that 33.7% of MS patients were significantly altered. Verbal memory (learning and recall), visuospatial long-term memory, working memory, and executive functions (DKEFS and COWAT) were the cognitive functions mostly impaired in MS patients compared to HC. As shown in Table 2, some cognitive tests (namely, COWAT, DKEFS DS, and CVLT LTM) were more sensitive to detect differences between the RRMS and HC groups.

In ND (EDSS ≤ 1.5), an unsatisfactory cognitive performance was limited to verbal memory and executive functions, while VMD patients (2 ≤ EDSS ≤ 2.5) also performed badly in information processing speed and visuospatial long-term memory. Overall, the VMD patients received lower scores than the ND patients in almost every test considered (Figure 3). Finally, MS patient showed higher levels of depression than HC (BDI score). Depression mood can affect cognitive performance; for this purpose, we applied ANCOVA with diagnosis and sex as factors and BDI as a covariate to compare cognitive performance of MS patients and HC. In general, all cognitive differences between MS patients and healthy controls still remained statistically significant.

These findings highlight and confirm that even considering very mildly clinically disabled MS patients, almost half of them experience some degree of cognitive impairment, suggesting that cognitive dysfunction can occur early in the disease. Particularly, executive functions and verbal memory can be impaired even before the onset of significant disability and can remain stable in VMD patients. In addition, information processing speed and visual memory are relatively preserved in ND patients and tend to deteriorate in the VMD group (see neuropsychological profiles shown in Figure 2).

Clinical disability generally progresses over the course of MS [4], although the correlation between cognitive impairment, clinical disability, and disease duration seems to be weak. Some studies found a neuropsychological performance impairment in recently diagnosed patients [24, 25], in patients with CIS [7, 26, 27], and in RRMS patients at early stages of the disease with little or no disability [7, 22, 26, 28]. Otherwise, many studies reported a poorer neuropsychological performance in patients with chronic progressive or secondary progressive MS [1, 6, 7, 9] than in RRMS patients. These findings imply that the higher the disability in a more advanced stage of MS, the greater the cognitive impairment. However, only a few studies investigated cognitive functioning in very mildly clinically disabled patients so far. Lynch and colleagues [16] showed a significant but slight association between cognitive impairment and clinical disability, independent of disease duration.

In the present study, disease duration ranged from three months to 30 years, but it did not significantly correlate with NP test scores. On the contrary, a significant correlation between clinical disability and CI was observed both in patients without disability and in patients with VMD. These findings, according to the literature [7, 8, 22, 26, 28], emphasize the existence of CI even in patients lacking clinical disability or in cases of VMD and show the progressive impairment of different cognitive domains related to EDSS worsening.

Furthermore, our results confirm the primary engagement of verbal memory and executive functions in very mild levels of clinical disability in accordance with previous studies [7, 29, 30]. Cerezo García and colleagues [29], in a small cohort of patients, found that 24% of very mild RRMS patients had memory deficits and 80% showed information processing speed and executive function impairment, especially in the maintenance of nonautomatic strategies and conceptual/categorization tasks, usually attributed to prefrontal regions. Roca and colleagues [30] showed that MS patients with low physical disability presented a fronto-subcortical pattern with impairment in memory, decision-making, working memory, and planning, as well as in goal-oriented behavior. This pattern correlated with loss of tissue integrity and organization in fronto-subcortical fiber tracts, particularly in the fronto-lateral (FL) areas, as measured with magnetic resonance diffusion tensor imaging. Interestingly, FL areas were specifically linked to executive cognitive dysfunction, such as poor planning, loss of inhibitory control, strategy development, cognitive flexibility, and working memory [31]. Ruano and colleagues [7], in a large Italian multicenter study, show a significant presence of CI since the earlier stages of MS in patients with RRMS and CIS with a more frequent involvement of information processing speed and executive function compared with other cognitive domains. In particular, these studies [7, 29, 30] used a specific executive battery consisting of sensitive tests to detect prefrontal cortex dysfunction.

Memory and executive impairment is relevant functions of the cognitive profile observed in MS and closely linked to prefrontal cortex functions. Memory is the process in which information is encoded, stored, and retrieved. Executive functions refer to the cognitive abilities needed for complex goal-directed behavior and adaptation to environmental changes and include several functions (working memory, reasoning, task flexibility, problem solving, and planning) [32]. Memory and executive function impairment negatively influences a patient's quality of life, as well as their everyday life functioning [33]. Nonetheless, in clinical practice, executive deficits are often misunderstood because their detection and characterization are not easy. Patients do not often complain about them, and the most used NP tests do not include specific executive functioning tests. Our study highlighted that an Italian version of MACFIMS is effective in assessing cognitive functioning in MS patients with very mild disability. This analysis method is specific, reliable, quick, and sensitive for the complete and comprehensive assessment of cognitive function in MS [3].

The high percentage (51.1%) of cognitive impairment in our sample of MS patients with very mild clinical disability could be due to employment of a complete and comprehensive neuropsychological analysis. It would be useful in clinical practice to use reliable and sensitive tools in order to early detect executive function impairment and to suggest an adequate cognitive training. The lack of a significant difference in the cognitive performance of the two patient groups can be attributed to the small VMD sample (*n* = 19). Future studies should therefore take into account the possibility of increasing the sample to evaluate possible differences. Another limitation of this study was the lack of a measure to assess fatigue and nutritional life style, two factors possibly influencing cognitive performance. Moreover, comparing different groups of patients, cognitive reserve is another important variable to be considered in future studies. Finally, another limitation could be the effect of interferon and glatiramer acetate on cognitive functioning; it has been demonstrated that some aspects of cognitive functioning may improve in patients with MS [34–36] and this may have had an impact on our results.

In conclusion, our study showed that half of our MS patients had an impaired performance on at least two cognitive tests, confirming that CI is a common symptom of MS even among patients with very mild or no clinical disability. Furthermore, compared to HC, the very mildly clinically disabled RRMS patients showed impairment of memory and executive functions with a main involvement of prefrontal cognitive functions. These results support the hypothesis that frontal lobes are highly sensitive, even in the early stage of the disease, due to their numerous connections with other cortical and subcortical regions, so that damages in any part of the brain can trigger effects in these areas [37, 38]. Memory impairment is probably related more to a failure in executive functioning, and in particular, organization and use of self-generated strategies to encode and recall new material could be less efficient, reflecting a poor performance of prefrontal functions. Detecting early executive dysfunctions with specific NP tests could be useful in order to promptly enroll MS patients in adequate rehabilitation projects.

## Supplementary Material

Table 4: Estimated means (standard error, SE) by ANCOVA model.

## Figures and Tables

**Table 1:** Demographic and clinical characteristics of the study sample.

	MS patients (92)	HC (42)	*p*
Age years (mean, SD)	41.5 (10.7)	42.0 (9.8)	0.766
Education, years [median (min–max)]	13.0 (5–18)	13.0 (8–18)	0.633
Women—*N* (%)	64 (69.6)	28 (66.7)	0.737
EDSS [median (min–max)]	1.0 (0–2.5)		
(i) EDSS ≤ 1.5: ND—*N* (%)	73 (79.3%)		
(ii) 2 ≤ EDSS ≤ 2.5: VMD—*N* (%)	19 (20.7%)		
Disease duration, years [median (min–max)]	9.5 (0.3–30.1)		

MS = multiple sclerosis patients; HC = healthy control subjects; ND = no physical disability; VMD = very mild disability.

**Table 2:** Neuropsychological tests scores.

Test		RRMS (*n* = 92)	HC (*n* = 42)	*p*	Cohen's d
CVLT TL	Mean (SD)	49.8 (9.6)	54.6 (9.3)	0.008	0.50
	% ± (mean-1.5 SD)	7.6%			
	% ± 5th percentile	5.4%			

CVLT LTM	Mean (SD)	−0.40 (1.06)	0.32 (0.92)	<0.001	0.70
	% ± (mean-1.5 SD)	18.5%			
	% ± 5th percentile	9.8%			

BVMT TL	Mean (SD)	45.6 (13.7)	47.9 (13.3)	0.372	0.17
	% ± (mean-1.5 SD)	27.2%			
	% ± 5th percentile	23.9%			

BVMT LTM	Mean (SD)	49.9 (13.2)	54.9 (10.3)	0.035	0.43
	% ± (mean-1.5 SD)	16.3%			
	% ± 5th percentile	14.1%			

PASAT 3″	Mean (SD)	38.7 (14.1)	41.9 (10.1)	0.187	0.25
	% ± (mean-1.5 SD)	22.8%			
	% ± 5th percentile	20.7%			

PASAT 2″	Mean (SD)	26.3 (14.0)	32.4 (13.2)	0.019	0.45
	% ± (mean-1.5 SD)	23.9%			
	% ± 5th percentile	19.6%			

SDMT	Mean (SD)	42.1 (11.2)	45.2 (6.4)	0.094	0.31
	% ± (mean-1.5 SD)	29.3%			
	% ± 5th percentile	19.6%			

DKEFS CS	Mean (SD)	8.2 (2.2)	9.8 (3.3)	0.001	0.62
	% ± (mean-1.5 SD)	10.9%			
	% ± 5th percentile	10.9%			

DKEFS DS	Mean (SD)	8.17 (2.6)	10.2 (3.3)	<0.001	0.73
	% ± (mean-1.5 SD)	13.0%			
	% ± 5th percentile	13.0%			

JLO	Mean (SD)	23.5 (4.5)	23.6 (5.1)	0.822	0.04
	% ± (mean-1.5 SD)	19.6%			
	% ± 5th percentile	17.4%			

COWAT	Mean (SD)	29.2 (11.1)	39.1 (13.6)	<0.001	0.83
	% ± (mean-1.5SD)	20.7%			
	% ± 5th percentile	17.4%			

BDI	Mean (SD)	13.0 (9.7)	5.1 (5.1)	<0.001	0.92
STAI-Y state	Mean (SD)	0.40 (1.25)	−0.09 (0.77)	0.188	0.44
STAI-Y state	Mean (SD)	−0.09 (1.03)	−0.31 (0.56)	0.474	0.24

CVLT TL: California Verbal Learning Test total learning; CVLT LTM. CVLT long-term memory; BVMT TL: Brief Visuospatial Memory Test total learning; BVMT LTM: BVMT long-term memory; SDMT: Symbol Digit Modalities Test; PASAT 3″ and 2″: Paced Auditory Serial Addition Test; JLO: Judgment of Line Orientation; DKEFS CS: Delis-Kaplan Executive Function System sorting test correct sort; DKEFS DS: DKEFS description score; COWAT: Controlled Oral Word Association Test; BDI: Beck Depression Inventory; STAI-Y: State-Trait Anxiety Inventory Form Y; RRMS: relapsing-remitting multiple sclerosis patients; HC: healthy controls. All neuropsychological tests were converted into a standard score using normative data.

**Table 3:** Post hoc comparison results. Data are presented as mean (SD); comparisons between VMD and ND are not showed, all *p* values > 0.2. Tukey's adjustment is applied (∗ indicates Dunnett's adjustment).

CVLT TL	Controls 54.6 (9.3)	Versus	ND 50.2 (9.1) *p* = 0.05
		Versus	VMD 48.2 (11.5) *p* = 0.045

CVLT LTM	Controls 0.3 (0.9)	Versus	ND −0.3 (1.0) *p* = 0.007
		Versus	VMD −0.7 (1.2) *p* = 0.001

BVMT TL	Controls 47.9 (13.3)	Versus	ND 47.1 (12.8) *p* = 0.946
		Versus	VMD 40.1 (15.6) *p* = 0.094

BVMT LTM	Controls 54.9 (10.3)	Versus	ND 51.2 (12.7) *p* = 0.265
		Versus	VMD 45.4 (14.2) *p* = 0.016

PASAT 3″	Controls 41.9 (10.1)	Versus	ND 39.5 (13.7) *p* = 0.597
		Versus	VMD 35.7 (15.8) *p* = 0.2

PASAT 2″	Controls 32.4 (13.2)	Versus	ND 26.5 (14.1) *p* = 0.075
		Versus	VMD 25.6 (14.2) *p* = 0.182

SDMT	Controls 45.2 (6.4)	Versus	ND 43.1 (11.2) *p* = 0.474^∗^
		Versus	VMD 38.3 (10.6) *p* = 0.042^∗^

DKEFS CS	Controls 9.8 (3.3)	Versus	ND 8.4 (2.2) *p* = 0.014
		Versus	VMD 7.5 (2.4) *p* = 0.005

DKEFS DS	Controls 10.2 (3.3)	Versus	ND 8.3 (2.6) *p* = 0.002
		Versus	VMD 7.6 (2.6) *p* = 0.003

JLO	Controls 23.6 (5.1)	Versus	ND 23.6 (4.8) *p* = 0.999
		Versus	VMD 22.8 (3.3) *p* = 0.813

COWAT	Controls 39.1 (13.6)	Versus	ND 30.2 (11.5) *p* = 0.002
		Versus	VMD 25.4 (8.7) *p* < 0.001

## References

[1] Chiaravalloti N. D., DeLuca J. (2008). Cognitive impairment in multiple sclerosis. *The Lancet Neurology*.

[2] Kalmar J. H., Gaudino E. A., Moore N. B., Halper J., DeLuca J. (2008). The relationship between cognitive deficits and everyday functional activities in multiple sclerosis. *Neuropsychology*.

[3] Benedict R. H., Cookfair D., Gavett R. (2006). Validity of the minimal assessment of cognitive function in multiple sclerosis (MACFIMS). *Journal of the International Neuropsychological Society*.

[4] Cook S. D. (2001). *Handbook of Multiple Sclerosis*.

[5] Migliore S., Ghazaryan A., Simonelli I. (2016). Validity of the minimal assessment of cognitive function in multiple sclerosis (MACFIMS) in the Italian population. *Neurological Sciences*.

[6] Denney D. R., Sworowski L. A., Lynch S. G. (2005). Cognitive impairment in three subtypes of multiple sclerosis. *Archives of Clinical Neuropsychology*.

[7] Ruano L., Portaccio E., Goretti B. (2016). Age and disability drive cognitive impairment in multiple sclerosis across disease subtypes. *Multiple Sclerosis Journal*.

[8] Langdon D. W. (2011). Cognition in multiple sclerosis. *Current Opinion in Neurology*.

[9] Amato M. P., Ponziani G., Siracusa G., Sorbi S. (2001). Cognitive dysfunction in early-onset multiple sclerosis: a reappraisal after 10 years. *Archives of Neurology*.

[10] Kujala P., Portin R., Ruutiainen J. (1997). The progress of cognitive decline in multiple sclerosis. A controlled 3-year follow-up. *Brain*.

[11] Schulz D., Kopp B., Kunkel A., Faiss J. H. (2006). Cognition in the early stage of multiple sclerosis. *Journal of Neurology*.

[12] Nocentini U., Pasqualetti P., Bonavita S. (2006). Cognitive dysfunction in patients with relapsing-remitting multiple sclerosis. *Multiple Sclerosis*.

[13] Gaudino E. A., Chiaravalloti N. D., DeLuca J., Diamond B. J. (2001). A comparison of memory performance in relapsing–remitting, primary progressive and secondary progressive, multiple sclerosis. *Cognitive and Behavioral Neurology*.

[14] Rao S. M., Leo G. J., Ellington L., Nauertz T., Bernardin L., Unverzagt F. (1991). Cognitive dysfunction in multiple sclerosis. II. Impact on employment and social functioning. *Neurology*.

[15] Amato M. P., Portaccio E., Stromillo M. L. (2008). Cognitive assessment and quantitative magnetic resonance metrics can help to identify benign multiple sclerosis. *Neurology*.

[16] Lynch S. G., Parmenter B. A., Denney D. R. (2005). The association between cognitive impairment and physical disability in multiple sclerosis. *Multiple Sclerosis*.

[17] Polman C. H., Reingold S. C., Banwell B. (2011). Diagnostic criteria for multiple sclerosis: 2010 revisions to the McDonald criteria. *Annals of Neurology*.

[18] Kurtzke J. F. (1983). Rating neurologic impairment in multiple sclerosis an expanded disability status scale (EDSS). *Neurology*.

[19] Benedict R. H., Fischer J. S., Archibald C. J. (2002). Minimal neuropsychological assessment of MS patients: a consensus approach. *The Clinical Neuropsychologist*.

[20] Beck A. T., Steer R. A., Ball R., Ranieri W. F. (1996). Comparison of Beck Depression Inventories-IA and-II in psychiatric outpatients. *Journal of Personality Assessment*.

[21] Spielberger C. D., Gorsuch R. L. (1983). *State-Trait Anxiety Inventory for Adults, Sampler Set: ManualTekst Booklet and Scoring Key*.

[22] Amato M. P., Portaccio E., Goretti B. (2010). Cognitive impairment in early stages of multiple sclerosis. *Neurological Sciences*.

[23] Cohen J. (1992). A power primer. *Psychological Bulletin*.

[24] Hankomäki E., Multanen J., Kinnunen E., Hämäläinen P. (2014). The progress of cognitive decline in newly diagnosed MS patients. *Acta Neurologica Scandinavica*.

[25] Lyon-Caen O., Jouvent R., Hauser S. (1986). Cognitive function in recent-onset demyelinating diseases. *Archives of Neurology*.

[26] Achiron A., Barak Y. (2003). Cognitive impairment in probable multiple sclerosis. *Journal of Neurology, Neurosurgery & Psychiatry*.

[27] Potagas C., Giogkaraki E., Koutsis G. (2008). Cognitive impairment in different MS subtypes and clinically isolated syndromes. *Journal of the Neurological Sciences*.

[28] Amato M. P., Zipoli V., Goretti B. (2006). Benign multiple sclerosis. *Journal of Neurology*.

[29] Cerezo García M., Martín Plasencia P., Aladro B. Y. (2015). Alteration profile of executive functions in multiple sclerosis. *Acta Neurologica Scandinavica*.

[30] Roca M., Torralva T., Meli F. (2008). Cognitive deficits in multiple sclerosis correlate with changes in fronto-subcortical tracts. *Multiple Sclerosis*.

[31] Cummings J. L. (1993). Frontal-subcortical circuits and human behavior. *Archives of Neurology*.

[32] Elliott R. (2003). Executive functions and their disorders: imaging in clinical neuroscience. *British Medical Bulletin*.

[33] Kleeberg J., Bruggimann L., Annoni J. M., Melle G., Bogousslavsky J., Schluep M. (2004). Altered decision-making in multiple sclerosis: a sign of impaired emotional reactivity?. *Annals of Neurology*.

[34] Lacy M., Hauser M., Pliskin N., Assuras S., Valentine M. O., Reder A. (2013). The effects of long-term interferon-beta-1b treatment on cognitive functioning in multiple sclerosis: a 16-year longitudinal study. *Multiple Sclerosis Journal*.

[35] Mokhber N., Azarpazhooh A., Orouji E. (2014). Cognitive dysfunction in patients with multiple sclerosis treated with different types of interferon beta: a randomized clinical trial. *Journal of the Neurological Sciences*.

[36] Mori F., Kusayanagi H., Buttari F., Nicoletti C. G., Bernardi G., Centonze D. (2012). Glatiramer acetate reverses plasticity and cognitive deficits associated with acute inflammation in MS (P04. 118). *Neurology*.

[37] Goldberg E. (1992). Introduction: the frontal lobes in neurological and psychiatric conditions. *Cognitive and Behavioral Neurology*.

[38] Goldberg E. (2002). *The Executive Brain: Frontal Lobes and the Civilized Mind*.

