# Supplementary material for: Cognitive Impairment in Relapsing-Remitting Multiple Sclerosis Patients with Very Mild Clinical Disability
Source: Behav Neurol. 2017 Aug 15;2017:7404289. doi: 10.1155/2017/7404289 (PMC5574272; doi:10.1155/2017/7404289)
Supplement: Supplementary file 1 — Table 4: Estimated means (standard error, SE) by ANCOVA model. [file 7404289.f1.docx]

**Table 4**: Estimated means (standard error, SE) by ANCOVA model.

| **TEST** | **RRMS** | **HC** | **P** | **Cohen’s d** |
| --- | --- | --- | --- | --- |
|  | **(n=92)** | **(n=42)** |  |  |
| **CVLT_TL** | 49.4 (1.36) | 54.3 (1.76) | 0.03 | 0.39 |
| **CVLT_LTM** | -0.45 (0.15) | 0.44 (0.19) | <0.001 | 0.65 |
| **BVMT_TL** | 43.97 (1.87) | 46.69 (2.43) | 0.376 | 0.16 |
| **BVMT_LTM** | 47.26 (1.8) | 53.35 (2.34) | 0.042 | 0.37 |
| **PASAT 3”** | 36.58 (1.9) | 39.52 (2.46) | 0.347 | 0.16 |
| **PASAT 2”** | 26.24 (1.94) | 31.13 (2.52) | 0.128 | 0.25 |
| **SDMT** | 41.51 (1.46) | 45.18 (1.89) | 0.128 | 0.27 |
| **DKEFS_CS** | 8.57 (0.36) | 9.82 (0.47) | 0.038 | 0.38 |
| **DKEFS_DS** | 8.51 (0.4) | 10.18 (0.52) | 0.012 | 0.45 |
| **JLO** | 23.1 (0.69) | 23.22 (0.89) | 0.917 | 0.02 |
| **COWAT** | 28.27 (1.63) | 38.49 (2.11) | <0.001 | 0.68 |

CVLT_TL. California Verbal Learning Test_Total Learning; CVLT_LTM. CVLT_Long Term Memory; BVMT_TL. Brief Visuo-spatial Memory Test_Total Learning; BVMT_LTM. BVMT_Long Term Memory; SDMT. Symbol Digit Modalities Test; PASAT - 3” and 2”. Paced Auditory Serial Addiction Test; JLO. Judgment of Line Orientation; DKEFS_CS. Delis Kaplan Executive Function System–Sorting Test_Correct Sort; DKEFS_DS. DKEFS_Description Score; COWAT. Controlled Oral Words Association Test; BDI. Beck Depression Inventory; STAI-Y. State-Trait Anxiety Inventory Form Y.

RRMS = Relapsing-Remitting Multiple Sclerosis patients. HC = Healthy controls.
